# Identification of RyR2-PBmice and the effects of transposon insertional mutagenesis of the RyR2 gene on cardiac function in mice

**DOI:** 10.7717/peerj.6942

**Published:** 2019-05-16

**Authors:** Qianqian Wang, Chao Wang, Bo Wang, Qirui Shen, Leilei Qiu, Shuaijun Zou, Tao Wang, Guoyan Liu, Beilei Wang, Liming Zhang

**Affiliations:** 1 Department of Marine Biotechnology, Faculty of Naval Medicine, Navy Medical University, Shanghai, China; 2 School of Life Sciences, China Jiliang University, Hangzhou, China; 3 Department of Nuclear Medicine, Changhai Hospital, Navy Medical University, Shanghai, China

**Keywords:** RyR2, Piggybac, Cardiomyocyte, Mitochondrion, Ca^2+^

## Abstract

Ryanodine receptor 2 (RyR2) plays an important role in maintaining the normal heart function, and mutantions can lead to arrhythmia, heart failure and other heart diseases. In this study, we successfully identified a *piggyBac* translocated RyR2 gene heterozygous mouse model (RyR2-PBmice) by tracking red fluorescent protein (RFP) and genotyping PCR. Cardiac function tests showed that there was no significant difference between the RyR2-PBmice and corresponding wild-type mice (WTmice), regardless of whether they were in the basal state or injected with epinephrine and caffeine. However, the sarcoplasmic reticulum Ca^2+^ content was significantly reduced in the cardiomyocytes of RyR2-PBmice as assessed by measuring caffeine-induced [Ca^2+^]_i_ transients; the cardiac muscle tissue of RyR2-PBmice displayed significant mitochondrial swelling and focal dissolution of mitochondrial cristae, and the tissue ATP content in the RyR2-PBmice heart was significantly reduced. To further analyze the molecular mechanism behind these changes, we tested the expression levels of related proteins using RT-PCR and Western blot analyses. The mRNA level of RyR2 in RyR2-PBmice cardiac tissue decreased significantly compared with the WTmice, and the protein expression associated with the respiratory chain was also downregulated. These results suggested that the *piggyBac* transposon inserted into the RyR2 gene substantively affected the structure and function of mitochondria in the mouse cardiomyocytes, leading to disorders of energy metabolism.

## Introduction

Ryanodine receptors (RyRs), the calcium release channels located in the sarcoplasmic reticulum (SR), are composed of four subunits with a molecular weight of 565 kDa each (Meissner, 2017). RyRs can be divided into three subtypes: RyR1, RyR2 and RyR3, with different coding genes (Ogawa, 1994). RyR2 is mainly distributed in cardiac tissue and participates in the excitation-contraction coupling of cardiac muscle through calcium-induced calcium release (CICR) (Santulli et al., 2017). Excitation-contraction coupling and cardiac performance are mainly based on two mechanisms: (1) synchronized Ca^2+^ release during contraction; and (2) effective Ca^2+^ reuptake that ensure a good and robust termination of Ca^2+^-dependent contraction (Gambardella et al., 2018). The RyR2 interactome ensures the appropriate amplitude and kinetics of Ca^2+^ cycling, which is indispensable for cardiac contractility. Henceforth, RyR2 represents the central target of many pathways dysregulated in cardiac pathological conditions, including arrhythmia, heart failure, metabolic disorders. All of these conditions are accompanied by alterations in Ca^2+^ handling and subsequent impairment in contractility (Gambardella et al., 2018; Heijman et al., 2014).

A variety of pathogenic mutations in the RyR2 encoding gene have been detected in heart dysfunction patients. Studies have shown that mutations in RyR2 can cause catecholaminergic polymorphic ventricular tachycardia (CPVT), a heritable arrhythmogenic disease resulting in exertional syncope or sudden death (Priori et al., 2002; Priori et al., 2001). RyR2 mutations can also cause arrhythmogenic right ventricular cardiomyopathy (ARVC), which is characterized by fatty infiltration and fibrosis of the myocardium (Bauce et al., 2002; Tiso et al., 2001). In addition, sudden infant death syndrome (SIDS) and atrial fibrillation (AF) are related to abnormalities of the RyR2 gene, and increasing numbers of patients with RyR2 mutation have been found so far. Given the diversity of RyR2 mutation types and their associated disease phenotypes, there are likely many asymptomatic RyR2 mutation carriers that have not been identified. Therefore, the function of the RyR2 gene and its relationship with diseases deserve further investigations.

A transgenic animal model can be a vital instrument to discover and assess human diseases. At present, a few developed types of RyR2 knockout or mutant mice have greatly promoted the studies on the pathophysiological roles and therapeutic implications of RyR2. For instance, considerable progress has been made on the mechanism of heart-related diseases in mutant mice such as R4496C (Cerrone et al., 2005), R176Q (Kannankeril et al., 2006) and R2474S (Lehnart et al., 2008). To identify more functional information of RyR2 and its relationship with human diseases, however, novel RyR2 mutation technology is crucial. Recently, the *piggyBac* (PB) transposon system was reported as a useful tool for genetic manipulations, including transgenesis and insertional mutagenesis in mice and other vertebrates (Ding et al., 2005), and a large-scale insertional mutagenesis with the PB transposon was performed in mice at the Institute of Developmental Biology and Molecular Medicine (IDM), Fudan University in Shanghai, China (Sun et al., 2008). To further investigate the physiological functions of the RyR gene, we obtained a strain of PB transposon inserted RyR2 gene heterozygous mouse (RyR2-PBmice) from Fudan University. As RyR2 is a key molecule for calcium regulation in cardiomyocytes, abnormalities in its structure and function may directly affect intracellular calcium homeostasis and induce tissue damage. Therefore, we utilized the RyR2-PBmice to evaluate the effects of the PB transposon inserted into the RyR2 gene on cardiac functions, attempting to further understand the physiological dysfunction underlying RyR2-associated cardiomyopathies.

## Methods

### Ethics statement

This study was carried out in strict accordance with the Guide for the Care and Use of Laboratory Animals published by the US National Institutes of Health (NIH) (NIH Publication No. 8023, revised 1978). All animal experiments were approved by the Institutional Animal Care and Use Committee of the Navy Medical University (81673348, 1 March 2016). To minimize any suffering of the animals, animals were anesthetized with 1.5% isoflurane before the experiments, and the response of animals during the investigation was monitored to ensure adequate depth of anesthesia. For tissue collection, animals were sacrificed by CO_2_ asphyxiation. All animals were housed in a specific animal room on a 12-h light/dark cycle at 22 ± 2 °C and 50–60% humidity, with no more than five per cage.

### Animals and reagents

The *piggyBac* inserted into the RyR2 gene mouse model was provided by the Institute of Developmental Biology, Fudan University (Shanghai, China). The PB transposon was inserted in the first intron of RyR2 on mouse chromosome 13, ENSMUSG00000021313, and the direction of the insertion was opposite to the gene location. All the mice used in this study were in compliance with the Ethics Statement described above.

Collagenase type II was provided by Worthington Biochemical Corporation, USA. Fetal bovine serum was purchased from Gibco, USA. Fluo-4 AM was purchased from Molecular Probes, USA. RIPA lysis buffer was purchased from the Beyotime Institute of Biotechnology, China. Other general agents were obtained from Sangon Biotech, China.

### Identification of the RyR2-PBmice

Offspring of the mice with transposons inserted into the RyR2 gene were phenotypically identified by the red fluorescent protein (RFP), a reporter gene carried by RyR2-PBmice. First, we irradiated the mice under an ultraviolet lamp to estimate their genotype by observing the red fluorescence. Then, the RFP images of the isolated cardiomyocytes were obtained by scanning with an Olympus FV1000 confocal microscope at an excitation spectrum of 543 nm.

The RyR2-PBmice strain was further determined by genotyping PCR with the primer pairs GL, GR and PB. β-actin was used as the inner gene when the upstream and downstream primers were 5′-GGCTGTATTCCCCTCCATCG-3′ and 5′-CCAGTTGTACACAATGCCATGT-3′.

### Evaluation of cardiac function in mice

RyR2-PBmice and corresponding wild-type mice (WTmice) were assessed for their susceptibility to stimulation-induced ventricular tachyarrhythmia using electrocardiogram (ECG) and heart rate (HR) recordings. Briefly, the mice were lightly anesthetized with 1.5% isoflurane vapor and 95% O_2_. The anesthetized mice were placed on a heating pad (27 °C), and needle electrodes were inserted subcutaneously into the right upper limb and left lower abdomen for ECG recording (MPA2000; Alcott Biotech, China). The animal ECG was continuously monitored under anesthesia until the heart rate stabilized. Baseline ECG was recorded for 15 min. For induction of ventricular arrhythmias, the mice were subjected to intraperitoneal injection of a mixture of epinephrine (2 mg/kg) and caffeine (120 mg/kg). ECG and HR were continuously recorded for 30 min after the injection of epinephrine and caffeine.

After the completion of monitoring, the mouse blood sample was collected at the tip of the heart. After resting for half an hour in a 1.5 ml EP tube, the sample was centrifuged and the heart injury-related enzymes were measured with an automatic biochemical analyzer (AIRONE-200puls, Crony Company, Italy).

### Myocardial cell calcium capacity test by the confocal microscopy

Single cardiomyocytes were isolated from 3-month-old RyR2-PBmice and WTmice by enzymatic digestion (Fares et al., 1998). Briefly, the hearts were excised from heparinized and deeply anaesthetized mice, cannulated and mounted on a Langendorff apparatus. After a digest perfusion for 8–10 min with perfusion buffer (mM: 10 HEPES, 0.6 Na_2_HPO_4_, 113 NaCl, 4.7 KCl, 12 NaHCO_3_, 0.6 KH_2_PO_4_, 1.2 MgSO_4_ ⋅7H_2_O, 10 KHCO_3_, 30 taurine, 10 2,3-butanedine monoxime, 5.5 glucose, pH 7.46) containing 773 U/ml collagenase type II (Worthington, USA), the ventricular tissue was cut into small pieces and gently stirred in stopping buffer containing perfusion buffer, 10% fetal bovine serum and 12.5 µM CaCl_2_ for 10–15 min. Then, the upper cell suspension was transferred to a 25 ml beaker, and Ca^2+^ was reintroduced to a final concentration of 1 mM. The isolated cardiomyocytes at the bottom were added to glass coverslips precoated with 50 mg/ml laminin and loaded with 5 mM Fluo-4 AM (Molecular Probes, Eugene, OR, USA) in bath solution containing (in mM) 135 NaCl, 4 KCl, 2 CaCl_2_, 1 MgCl_2_ ⋅H_2_O, 10 HEPES, 1.2 NaH_2_PO_4_ ⋅H_2_O and 10 glucose for 20 min at room temperature. Following the monitoring of Ca^2+^ at rest, cells were excited by caffeine stimulation. Confocal line scanning (512 pixels and 1 ms per line) was performed along the longitudinal axis of cells for 10 s using an Olympus FV1000 confocal system (Olympus Corporation, Tokyo, Japan). The fluorescence values (F) were normalized by the basal fluorescence (F_0_) in order to obtain the fluorescence ratio (F/F_0_).

### Cardiac pathological examination

The mouse heart was excised and washed in ice-cold PBS (0.1 M phosphate) after evaluation of cardiac function. For histological analysis, the hearts were fixed with a 10% formalin solution and stained with hematoxylin and eosin for microscopy examination (ECLIPSE 55i Nikon, Tokyo, Japan). The heart samples for transmission electron microscopy observation were fixed in 1% potassium ferrocyanide reduced O_S_O_4_, dehydrated through graded ethanol, and coated with epoxy resin. Ultrathin slices were stained with saturated uranium acetate and silver citrate and viewed using a transmission electron microscope (H-600, Hitachi, Japan).

### Measurement of ATP in the heart tissue

We used a commercial kit (Beyotime Institute of Biotechnology, Shanghai, China) to quantify the content of adenosine triphosphate (ATP) according to the manufacturer’s protocol. In brief, the samples of heart tissues (each approximately 10 g) were homogenized and centrifuged at 1, 200 × g for 10 min at 4 °C. The supernatants were subjected to immediate measurement of ATP levels.

### RT-PCR and Western blotting

For the quantitative RT-PCR assay, the total RNA was isolated from the heart tissues of RyR2-PBmice and WTmice by using TRIzol reagent (Invitrogen, USA), and the iScript reaction (Bio-Rad, USA) was used to synthesize the cDNA. Quantitative RT-PCR with iTaq Universal SYBR Green Supermix (Bio-Rad, USA) was performed on a StepOne Real-Time PCR System (Applied Biosystems, USA) to determine gene expression using the relative standard-curve method. β-actin was used as an internal control. The primer sequence information is shown in Table 1 below.

**Table 1:** RT-PCR primer sequence information.

*Species: [Mus musculus]*					
No.	Gene Symbol	GenBank	Forward primer	Reverse primer	Product
1	Ryr2	NM_023868.2	GAGAAGCCATCCGAATTAGG	ATCTTTGGCTATTGTTGGCA	104
2	Gnb3	NM_013530.1	AAGCCACGAAGGTTCTCA	CAAAGGCACACTCCCATAAT	128
3	Cox8b	NM_007751.3	TAGTCGTTGGCTTCATGGTT	GAAATCTCTCAGGGATGTGC	101
4	Gpr37l1	NM_134438.3	TCTAAAGTGGGTCTGTCTTAGC	CACCTAACGGGAACAATGCAA	111
5	Cdh11	NM_009866.5	TGTGGATGTGGACGACTT	CGGCCCTCATAACCATAGA	102
6	Micu3	NM_030110.1	TTGAATGGAGTTTGTCAGCG	TGATTTAGTGTTTGCAGCAAGT	112
7	mt-Co2	NP_904331.1	CAGGCCGACTAAATCAAGCAA	AGTGGAACCATTTCTAGGACA	121
8	mt-Atp6	NP_904333.1	TCAACAACCGTCTCCATTCT	ATTAGGGTTCATGTTCGTCC	100
9	mt-Co3	NP_904334.1	CTATTCATCGTCTCGGAAGTAT	GTGAAATTCCTGTTGGAGGTC	115
10	Nppb	NM_001287348.1	GACCACCTTTGAAGTGATCC	TGTGGCAAGTTTGTGCTC	122
11	Ucp3	NM_009464.3	TCAGGGTGTTGGGAAGATAGAA	AACATTGTCCTCAGGCTTAC	110
12	ACTB	NM_007393.3	GGCTCCTAGCACCATGAAGA	AGCTCAGTAACAGTCCGCC	187

For Western blotting analysis, the total proteins were extracted from the frozen hearts with RIPA lysis buffer according to the manufacturer’s instructions and quantified with the BCA™ Protein Assay Kit (Bio-Rad, Hercules, CA, USA). Antibodies to RyR2 (1:1000, ab196335; Abcam, Cambridge, UK), RFP (1:1,000, ab62341; Abcam), GNB3 (1:2,000, ab154866; Abcam), COX (1:1,000, ab33985; Abcam), LGR5 (1:2,000, ab75732; Abcam), PMCA (1:1,000, ab2825; Abcam), Natriuretic Peptide Receptor B (NPPB, 1:2,000, ab139188; Abcam) and UCP (1:1,000, ab10985; Abcam) were used. Briefly, equal amounts of samples were subjected to SDS-PAGE and then transferred onto nitrocellulose membranes (Bio-Rad, Hercules, CA, USA). The membranes were blocked with Tris-buffered saline (TBS-T, 20 mM Tris-HCl (pH 7.4), 150 mM NaCl, 0.02% Tween 20) and then blocked with 5% nonfat milk for 1 h at room temperature. After this, the membranes were incubated in the appropriate primary antibodies overnight at 4 °C, followed by HRP-conjugated secondary antibodies (1:1,000, #5127; Cell Signaling Technology, Danvers, MA, USA) at room temperature for 1 h. To control equal loading of total protein in all lanes, blots were stained with rabbit anti-β-actin antibody (1:1,000, #4970; Cell Signaling Technology, Danvers, MA, USA) for equal loading of proteins. After repeated washing, membranes were incubated with an enhanced chemiluminescence system (ECL, Amersham) according to the manufacturer’s instructions followed by visualization with X-ray film. Quantitative analysis was carried out with NIH ImageJ software.

### Statistics

All the quantitative data are expressed as the mean ± SD. The graphs were built using GraphPad Prism 5 for each experiment. One-way analysis of variance (ANOVA) followed by Tukey’s post hoc test was used, and a statistical significance was indicated by *P* < 0.05.

## Results

### Identification of the RyR2-PBmice

**Figure 1:**
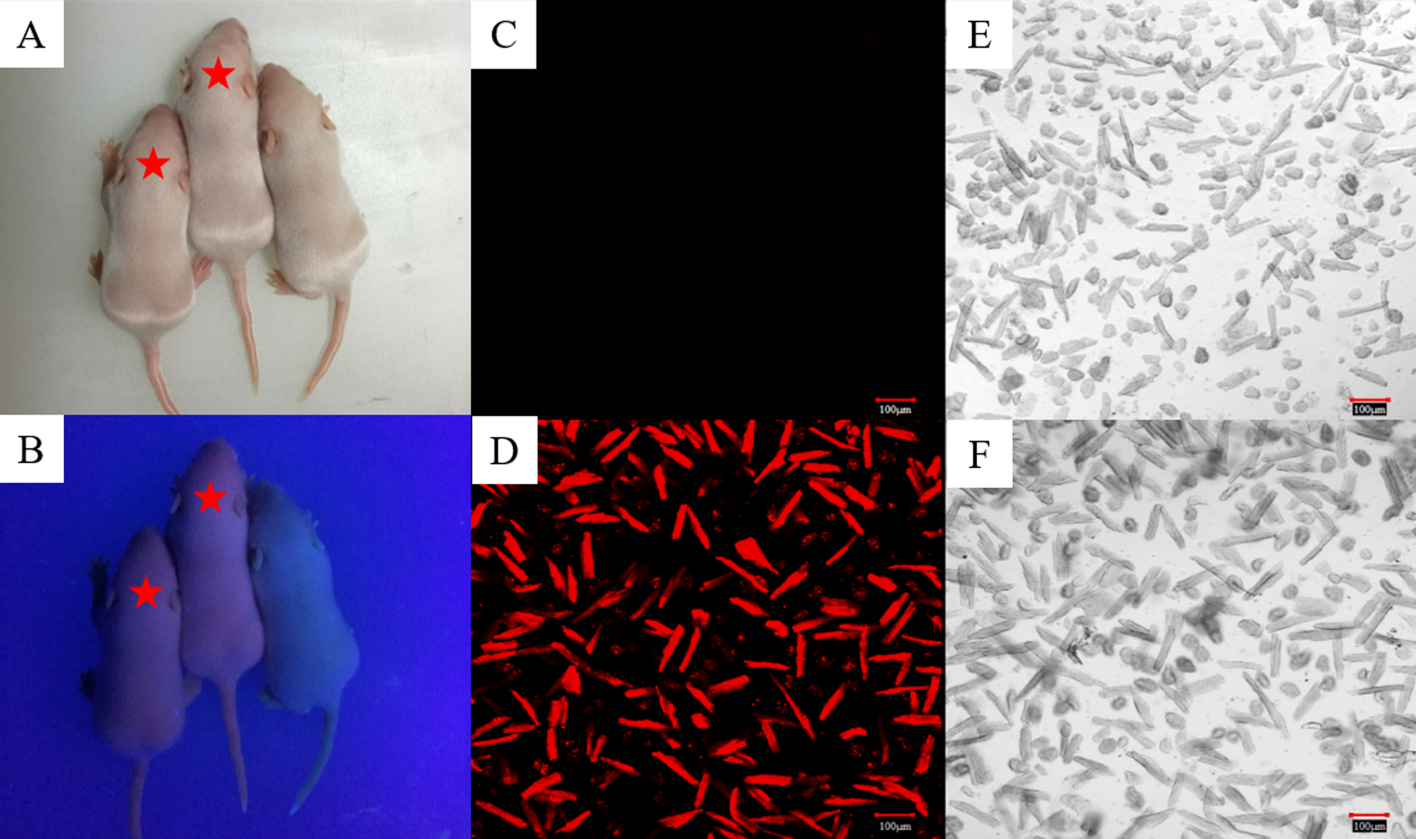
Identification of RyR2-PBmice phenotype by tracking RFP. The same litters of mice under the illumination of fluorescent (A) and ultraviolet (B) lamps are shown here. The asterisk marks the two heterozygous RyR2-PBmice in which red fluorescence is clearly visible under fluorescent illumination and the other one is a homozygous WTmice that does not fluoresce. (C) and (D): Images of the ventricular myocytes isolated respectively from WTmice and RyR2-PBmice under red fluorescence. (E) and (F): Images of the ventricular myocytes isolated respectively from WTmice and RyR2-PBmice in the white light.

Based on the information we obtained from the PBmice mutation database, the RyR2-PBmice were a strain of heterozygous mouse transposed by a PB transposon element with an RFP gene, so when illuminated by ultraviolet (UV) light, the red fluorescence could be observed in the heterozygous RyR2-PBmice. We found that all surviving individuals in the third generation of breeding showed the same appearance under a fluorescent lamp (Fig. 1A). However, the heterozygous RyR2-PBmice, but not homozygous WTmice, showed visible red fluorescence under UV illumination (Fig. 1B). The results observed in the isolated ventricular myocytes were consistent with those in vivo: the ventricular myocytes from RyR2-PBmice but not from WTmice showed red under RFP excitation.

**Figure 2:**
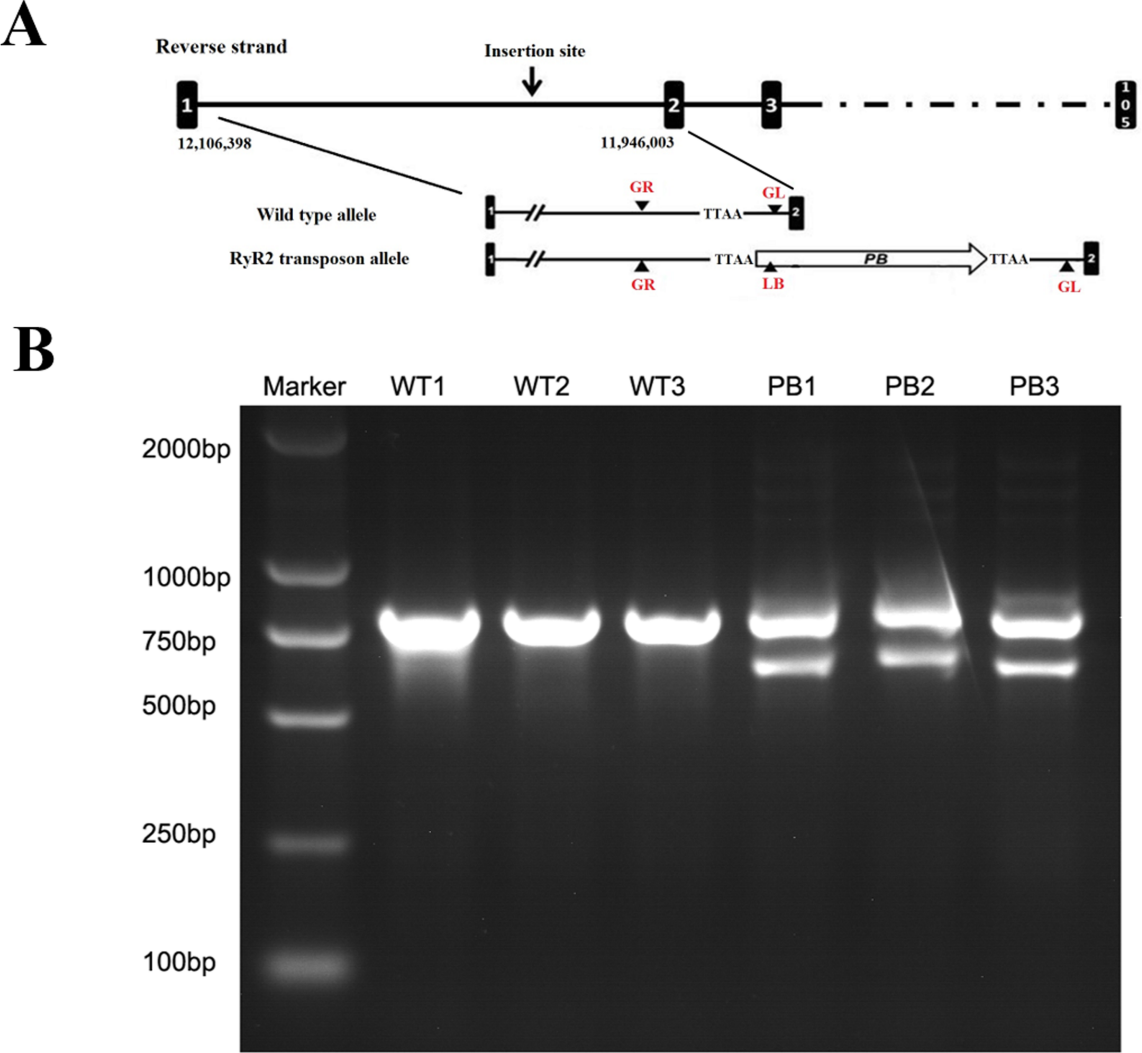
Characterization of the PB insertion in mice. (A) Position of the PB insertion. PB integration site was mapped to the first intron of RyR2 gene on mouse chromosome 13. Diagram shows the genomic structure of RyR2 gene with intron (lines) and exons (black boxes), with the gene specific primers indicated below. TTAA: the four nucleotides required for PB insertion. (B) Characterization of the PB insertion by PCR using the indicated primers LB, GL and GR. The different genes were determined by 3-primer PCR to detect WT (823 bp) and PB insertion alleles (655 bp and 823 bp).

The genotypes of WTmice and RyR2-PBmice were further identified with the PCR assay. In this procedure, an insertion line carrying the PB transposon was mapped in the RyR2 transposon allele (Fig. 2A). GL, GR and LB primers were used to explore the transposition behavior of PB in the mice. Due to the transposon insertion, the 655 bp and 823 bp PCR products were detected in the RyR2-PBmice, while only an 823 bp product was detected in the WTmice (Fig. 2B).

### Effects of transposon insertional mutagenesis in the RyR2 gene on cardiac function

**Figure 3:**
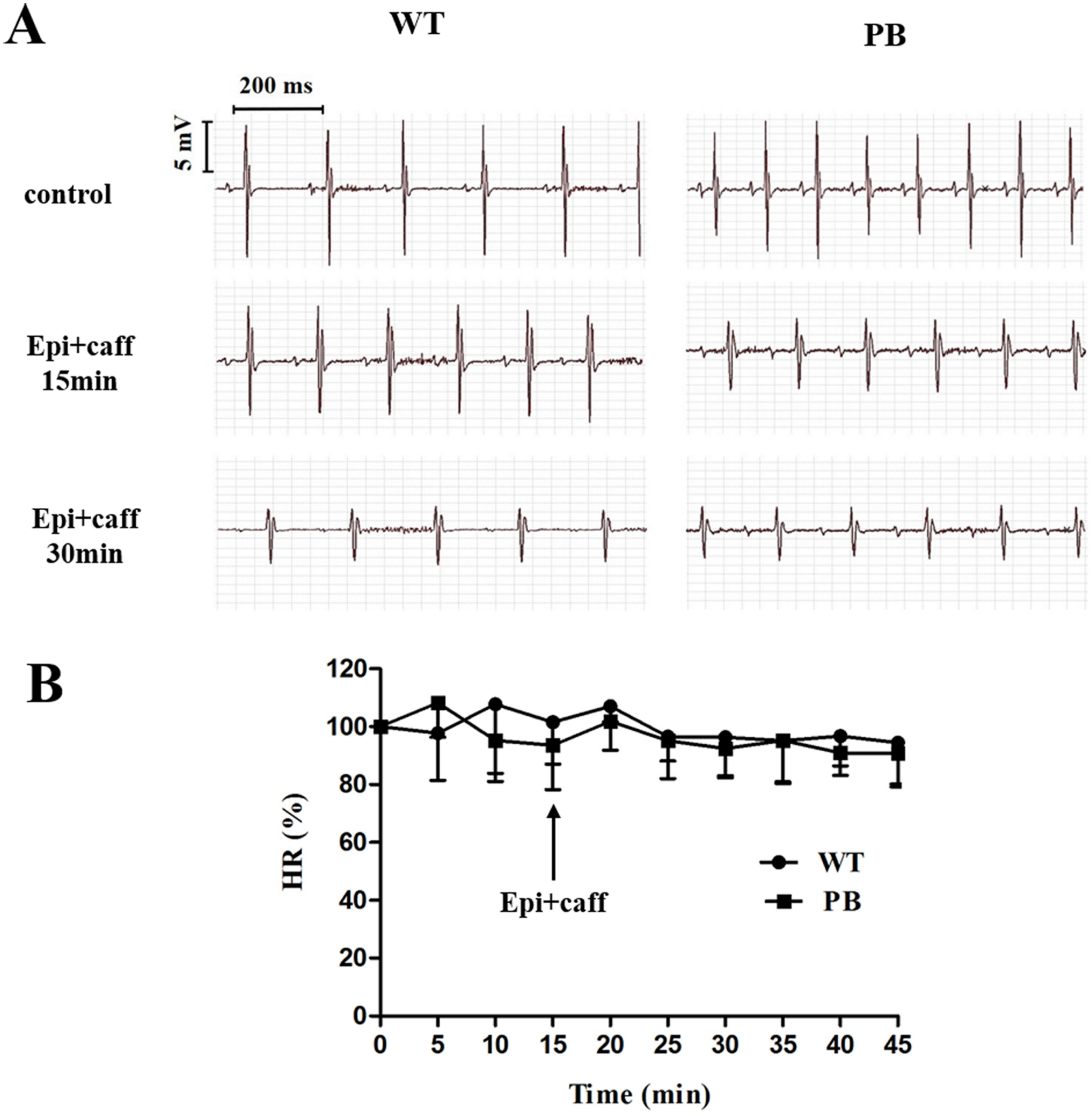
Representative ECG and HR of WTmice and RyR2-PBmice. (A) Representative ECG recordings of WTmice and RyR2-PBmice before and after the injection of epinephrine (2 mg/kg) and caffeine (120 mg/kg). (B) HR of WTmice and RyR2-PBmice ( *n* = 6).

**Figure 4:**
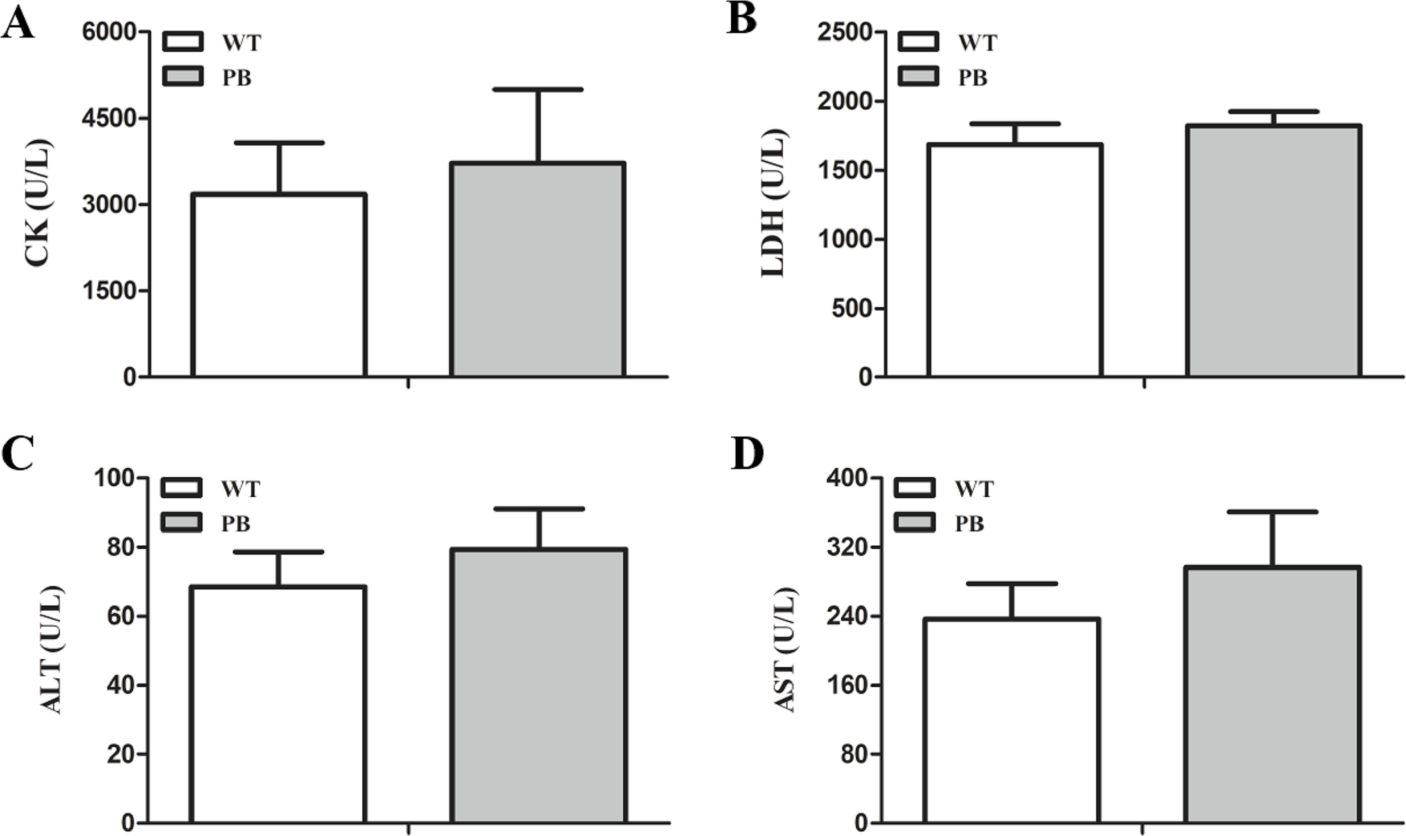
Changes in myocardial enzymes of WTmice and RyR2-PBmice. (A) CK: Phosphocreatine kinase. (B) LDH: lactate dehydrogenase. (C) ALT: Alanine aminotransferase. (D) AST: Aspartate aminotransferase.

We monitored the ECG and HR of the RyR2-PBmice before and after the injection of pharmacological triggers (caffeine and epinephrine) to assess the effects of insertional mutagenesis in the RyR2 gene by PB transposon on cardiac function. Unexpectedly, we found that caffeine and epinephrine did not induce abnormal cardiac function in either RyR2 mutant mice or their WT littermates at 2–3 months of age (Fig. 3). We further detected cardiac function-related enzymes to evaluate heart injury in RyR2-PBmice. Similarly, we did not find any significant difference in phosphocreatine kinase (CK), lactate dehydrogenase (LDH), alanine aminotransferase (ALT) and aspartate aminotransferase (AST) indexes between the RyR2-PBmice and WTmice (Fig. 4).

### Effects of PB insertion on intracellular Ca^2+^ homeostasis in cardiomyocytes

**Figure 5:**
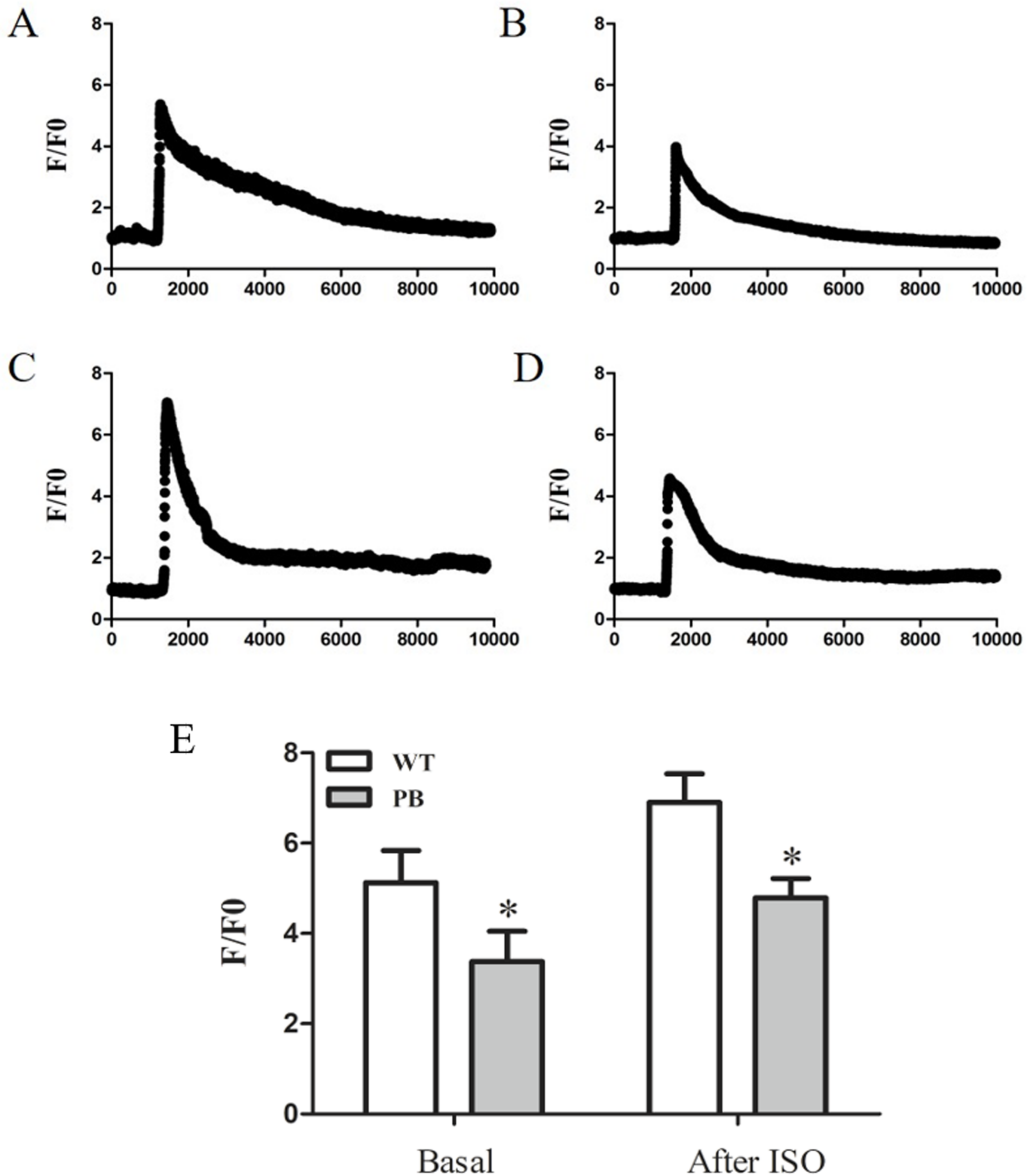
Ca^2+^ capacity in WTmice and RyR2-PBmice cardiomyocytes. Ventricular myocytes isolated from WTmice and RyR2-PBmice hearts were loaded with Fluo-4 AM, perfused with 1.8 mM extracellular Ca^2+^ in bath solution and stimulated with caffeine and ISO. Ca^2+^ transients were monitored by linescan confocal Ca^2+^ imaging. Representative traces of WTmice (A) and RyR2-PBmice (B) cardiomyocytes, and representative traces of WTmice (C) and RyR2-PBmice (D) cardiomyocytes after trigger ed by ISO are shown. Average value of the amplitude is shown in (E). (*n* = 6, * *P* < 0.05 *vs.* WTmice).

To evaluate the influence of PB insertion on intracellular Ca^2+^ homeostasis in RyR2-PBmice, we measured caffeine-evoked [Ca^2+^]_i_ transients using the Ca^2+^ indicator Fluo-4 AM. Figures 5A and 5B show the corresponding [Ca^2+^]_i_ transients by caffeine stimulation in cardiomyocytes obtained from WTmice and RyR2-PBmice, respectively. The amplitude of [Ca^2+^]_i_ transients (F/F0) was lower in RyR2-PBmice cardiomyocytes than in WTmice. We further measured the isoproterenol (ISO)-evoked [Ca^2+^]_i_ transients and found that the [Ca^2+^]_i_ capacity was also significantly reduced in the RyR2-PBmouse cardiomyocytes.

### Mitochondrial structure disorder in cardiomyocytes

**Figure 6:**
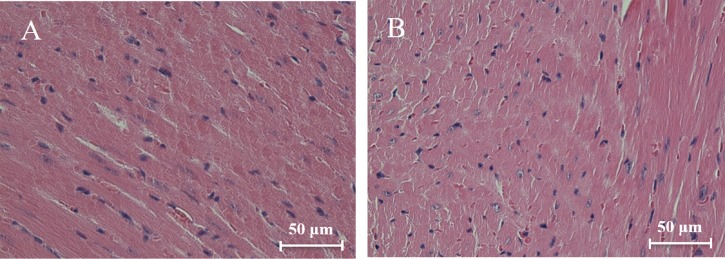
Histological analysis of heart sections (H&E staining). (A) WTmice; (B) RyR2-PBmice. Scale bars: 50 µm.

**Figure 7:**
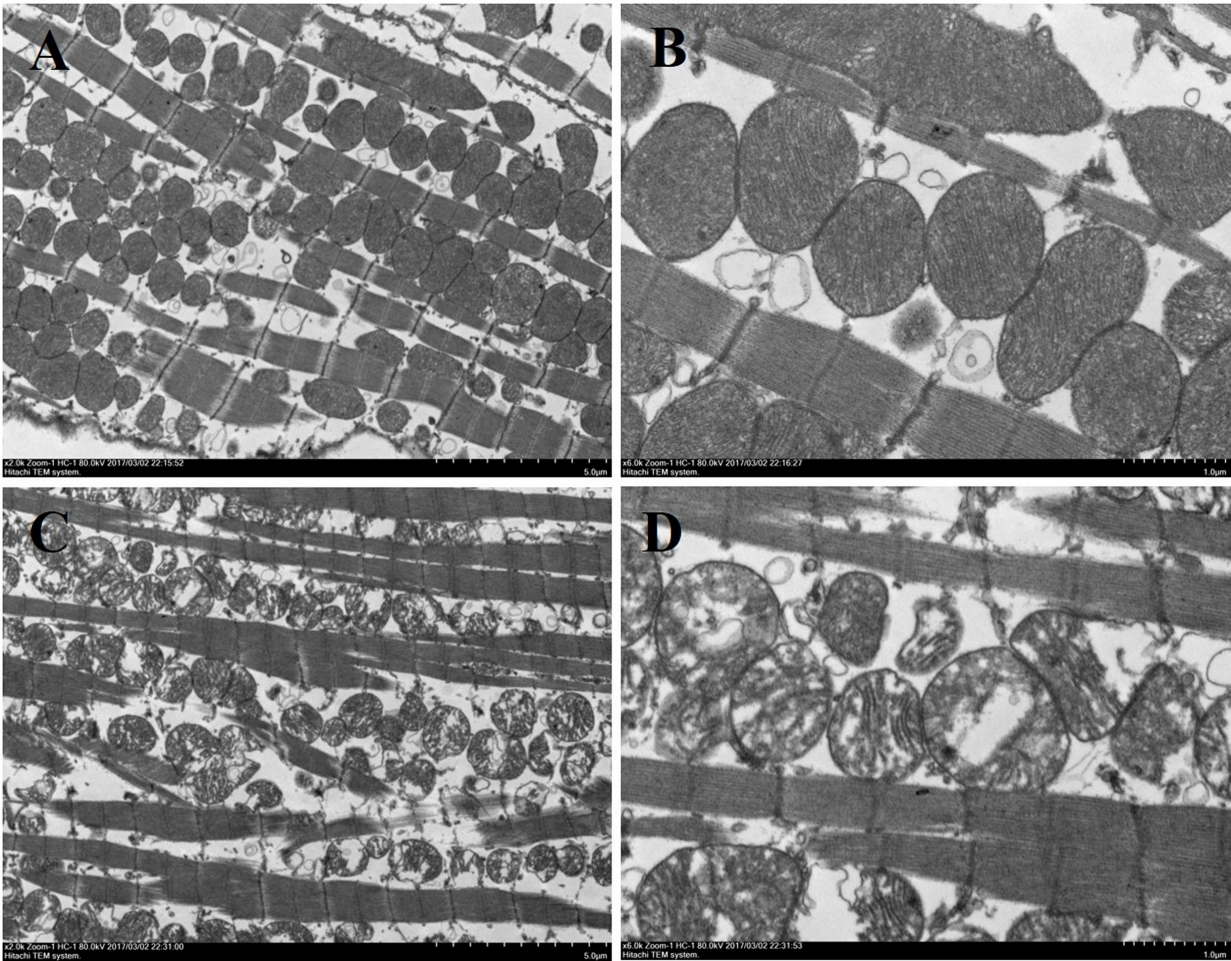
Ultrastructure of myocardial mitochondrial in transmission electron microscopy. (A) and (B): cardiac tissue of WTmice; (C) and (D): cardiac tissue of RyR2-PBmice. (*n* = 3, scale bars in A and C, 5 µm, scale bars in B and D, 1 µm)

Histological analysis of heart sections stained with hematoxylin and eosin revealed that the cardiac sarcomere was well-arranged and no obvious lesions were observed in the myocardium from both WTmice and RyR2-PBmice (Fig. 6). To investigate the ultrastructure of the mitochondria, transmission electron microscopy was performed on cardiac muscle tissue (Fig. 7). Compared with the WTmice, significant mitochondrial swelling and focal dissolution of mitochondrial cristae occurred in RyR2-PBmice. These results suggested that while no obvious histological changes occurred in the myocardial structure of RyR2-PBmice, PB transposons inserted into the RyR2 gene disrupted the ultrastructure of myocardial mitochondria.

### ATP concentration in heart tissues

**Figure 8:**
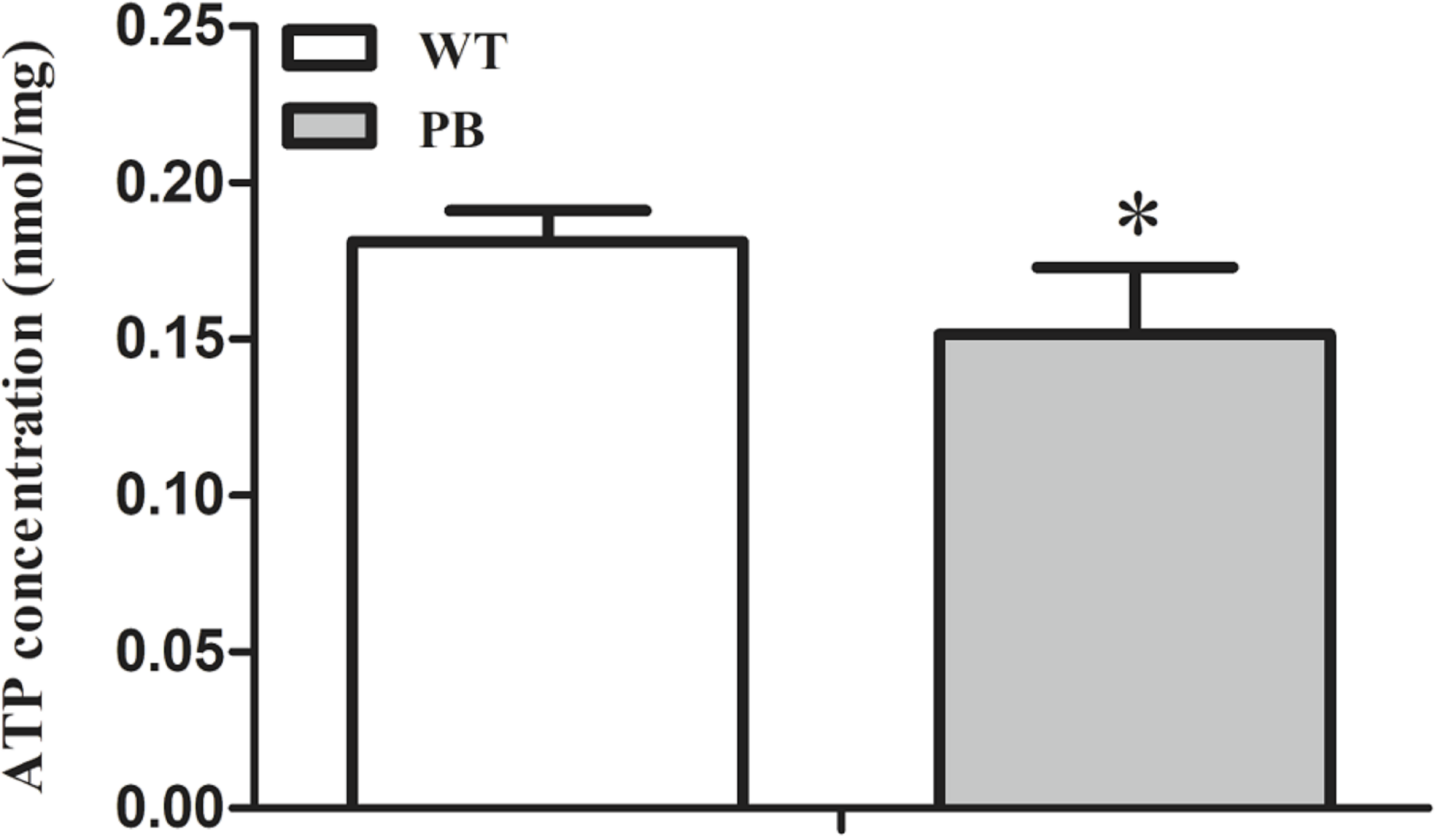
ATP concentration in myocardial tissue of WTmice and RyR2-PBmice. (*n* = 6, * *P* < 0.05 *vs.* WT).

To investigate whether the myocardial mitochondrial structure affected cardiac function, we examined the concentration of ATP in myocardial tissue to evaluate its energy supply. The results showed that the ATP concentration in RyR2-PBmouse heart tissue was significantly reduced compared with WTmice (Fig. 8), indicating that the *piggyBac* inserted into the RyR2 gene could interfere with the energy metabolism level of the mitochondria.

### RT-PCR and Western blotting

**Figure 9:**
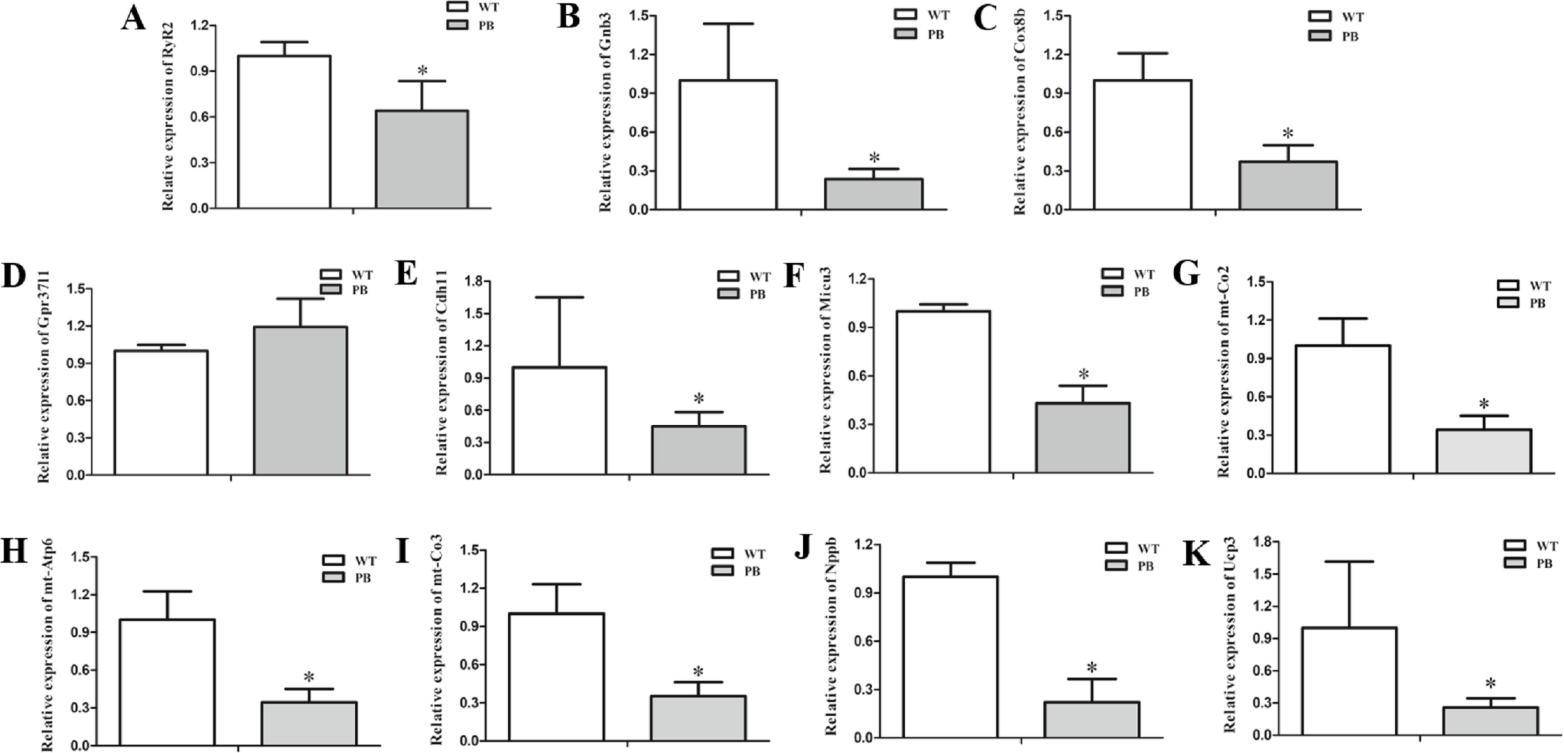
Quantitative RT-PCR analysis of RyR2 mRNA and the related signaling pathway coding sequences in WTmice and RyR2-PBmice. cDNAs from the heart tissue were amplified as templates; GAPDH was used as an internal control. (A) RyR2; (B) Gnb3; (C) Cox8b; (D) Gpr37l1; (E) Cdh11; (F) Micu3; (G) mt-Co2; (H) mt-Atp6; (I) mt-Co3; (J) Nppb; (K) Ucp3.

To examine the expression level of the RyR2 and genes from its related signaling pathway, real-time quantitative PCR (qPCR) was applied to quantify the 3′-end transcripts with primer pairs located on these genes. The mRNA levels of a variety of genes were decreased, including RyR2 (Fig. 9A), Gnb3 (Fig. 9B), Cox8b (Fig. 9C), Cdh11 (Fig. 9E), Micu3 (Fig. 9F), mt-Co2 (Fig. 9G), mt-Atp6 (Fig. 9H), mt-Co3 (Fig. 9I), Nppb (Fig. 9J) and Ucp3 (Fig. 9K), whereas the Gpr37l level was increased (Fig. 9D). Gnb3 and Gpr37 l1 are related to guanine nucleotide binding proteins, which are involved as modulators or transducers in various transmembrane signaling systems and are important in regulating calcium channels. Cox8b, Micu3, mt-Co2, mt-Atp6, mt-Co3 and Ucp3 are responsible for the oxidative phosphorylation and ATP synthesis in the mitochondrial. Nppb, the B type natriuretic peptide, is a hormone secreted by cardiomyocytes in the heart ventricles in response to stretching caused by increased ventricular blood volume. These genes are essential for normal cardiac and mitochondrial function, and the reduction of these genes at the transcriptional level also suggested that the insertion mutation of RyR2 could result in mitochondrial energy metabolic disorders.

**Figure 10:**
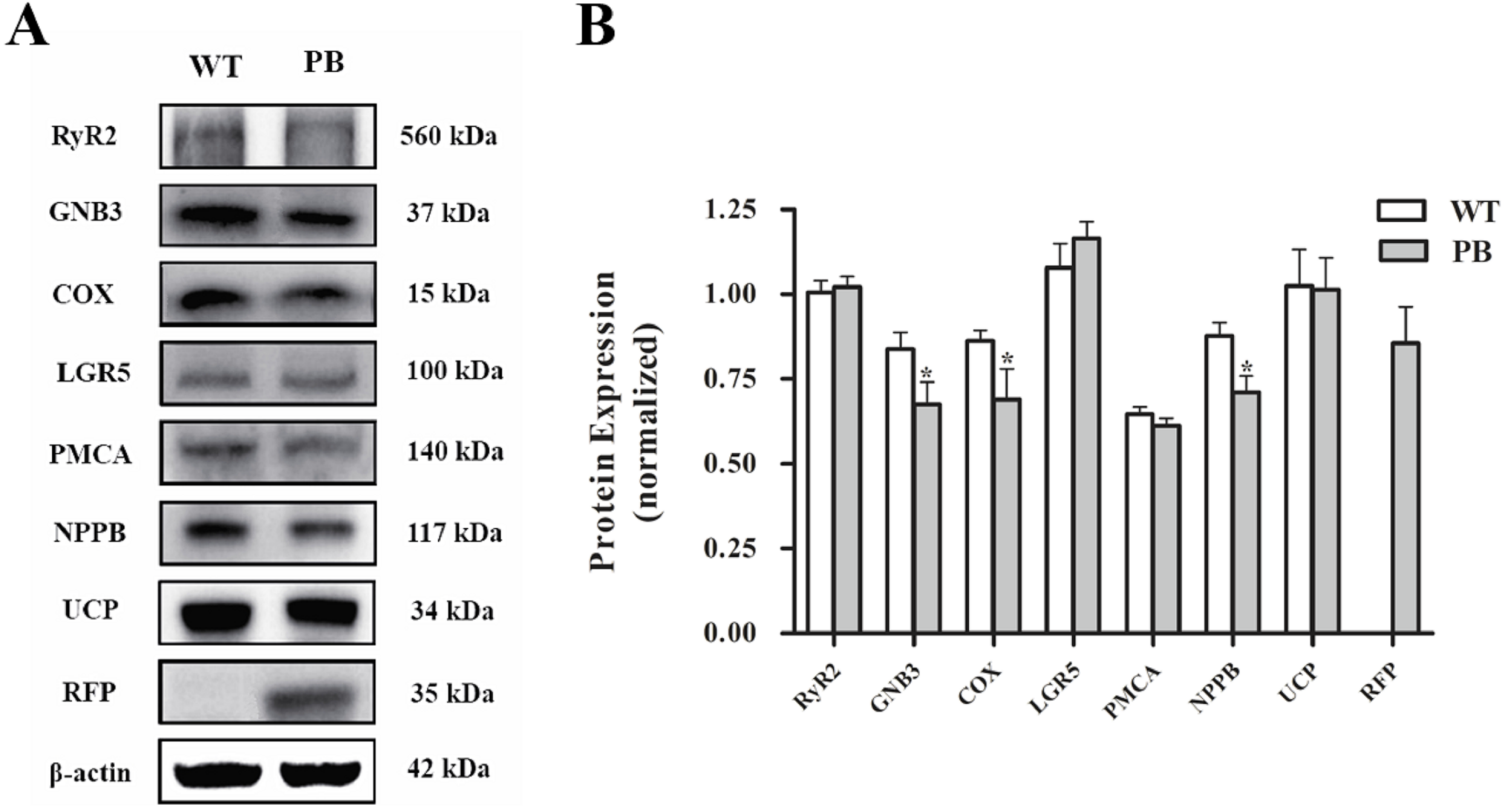
Western blot analysis of RyR2 and the related proteins regulating Ca ^2+^ and ATP concentration in the heart tissues. (A) Expression of the proteins in RyR2-PBmice and WTmice; (B) Relative density analysis of the protein bands. β-actin was probed as an internal control in relative density analysis of the protein bands. The relative density is expressed as the ratio (proteins/ β-actin). The vehicle control is set as 1.0. Each value is expressed as mean ± SD. WT, WTmice; PB, RyR2-PBmice. (*n* = 3, * *P* < 0.05 *vs.* WT).

Western blot analysis (Fig. 10) showed that there was no significant difference in the protein levels of RyR2, PMA and G protein-coupled receptors between WTmice and RyR2-PBmice, while the expression levels of small G protein and cytochrome C oxidase associated with the RyR2 signal pathway significantly decreased.

## Discussion

The RyR2 gene is one of the largest genes in the human genome and is closely associated with the cardiac structure and function. For the insertional mutagenesis model of RyR2-PBmice used in this study, the homozygous RyR2-PBmice were embryonically lethal, most likely due to the marked lesions resulting from the RyR2 mutation, while the heterozygous RyR2-PBmice showed no apparent differences compared with the corresponding WTmice.

The expression level of the RyR2 gene might be changed by PB insertion, leading to speculations as whether the heart pathologies would appear in the mutants. Therefore, we assessed cardiac function in RyR2-PBmice and found that the heterozygous RyR2-PBmice did not exhibit bradycardia or death. In most cases, both the RyR2-mutated patients and RyR2-mutated mice show non-organic heart disease (Liu et al., 2006; Santulli et al., 2018). This finding means that heart function in such cases is often normal under basic conditions. However, when the RyR2-mutated mice undergo exercise fatigue or epinephrine stimulation, spontaneous Ca^2+^ release disorders often cause arrhythmias (Cerrone et al., 2005; Lehnart et al., 2008; Liu et al., 2006). Therefore, we injected RyR2-PBmice with epinephrine and caffeine to further determine whether these stimulants could induce arrhythmias or other heart diseases. To our surprise and unlike the results observed in most RyR2 mutants, heterozygous RyR2-PBmice were not susceptible to stimulation-induced cardiac arrhythmias. We speculated that the maintenance of the clinical phenotypes observed in RyR2-PBmice is attributable to the variable expression levels of the RyR2 mutant allele.

However, it is crucial to know whether the transposon inserted into the RyR2 gene really maintains its “silence”. Previous studies have revealed that mutation of the RyR2 gene can cause abnormalities in the plasma network calcium leakage, resulting in abnormal heart function (Shan et al., 2012; Xie et al., 2013; Zhang et al., 2011). Li found that abnormal SR Ca^2+^ leakage via RyR2 is a molecular mechanism underlying atrial fibrillation (AF) progression and long-lasting spontaneous AF (sAF) development (Li et al., 2014). Multiple G1885E/G1886S mutations usually cause modification of the RyR2 channels, increase the free calcium concentration in the diastolic phase, and cause calcium leakage in SR, eventually triggering lethal arrhythmias (Wehrens et al., 2003). In addition, hereditary RyR2 mutations may result in sudden deaths, also due to the massive calcium leakage during overwork or adrenergic stimulation (Huang et al., 2014; Ran et al., 2010). At present, there are still controversies regarding the mechanism of RyR2 mutations leading to abnormal cardiac function. However, SR diastolic calcium leakage due to abnormal RyR2 channel function is the most important link in heart pathogenesis (Duan, 2010). Hence, we determined the Ca^2+^ transients in cardiomyocytes isolated from RyR2-PBmice and WTmice. Interestingly, we found that the SR Ca^2+^ capacity in RyR2-PBmice cardiomyocytes was significantly lower than that in WTmice, and this effect was magnified by isoproterenol stimulation. This result is different from the previous reports of RyR2 mutations resulting in SR Ca^2+^ leakage mechanisms (Jiang et al., 2005; Jiang et al., 2004). Bers found that the SR Ca^2+^ concentration [Ca^2+^]_SR_ had a dominant effect on the opening of RyR2. When [Ca^2+^]_SR_ increased, it induced the RyR2 channel to open, causing increased calcium leakage (Bers, 2002). In our study, the [Ca^2+^]_SR_ was reserved at a relatively low level and was not prone to high levels of calcium leakage. Therefore, to some extent, RyR2-PBmice inhibited the spontaneous release of calcium from RyR2, which might explain why RyR2-PBmice did not show calcium-disordered arrhythmias even under adrenergic stimulation conditions.

Nevertheless, we cannot completely rule out the potential effects of reduced [Ca^2+^]_SR_ on RyR2-PBmice. Cardiomyocyte [Ca^2+^]_SR_ directly controls the contractile properties of the heart, and the insufficient release of the SR Ca^2+^ store can result in a decrease in myocardial contractility. Although under the current conditions, we did not observe significant cardiac dysfunction in RyR2-PBmice, the lack of [Ca^2+^]_SR_ remains a potential risk and will always be present in the RyR2-PBmice.

However, the RyR2 mutation not only caused life-threatening arrhythmia but also affected the structure of the myocardium (Bhuiyan et al., 2007; Tiso et al., 2001). Recent studies have found that more than 9% of RyR2 mutants in ARVC cases result in structural heart disease (Roux-Buisson et al., 2014). Myocardial contraction requires RyR2 release of sufficient Ca^2+^ from the SR and mitochondria production of energy by producing ATP; thus, any abnormal situation that affects the Ca^2+^ release or ATP synthesis could cause heart-associated diseases (Borutaite, Toleikis & Brown, 2013). Since mitochondrial Ca^2+^ uptake via the mitochondrial Ca^2+^ uniporter is dependent on SR Ca^2+^ release, alterations in SR Ca^2+^ release can also affect mitochondrial function by regulating mitochondrial Ca^2+^ uptake. Recent studies have shown that endoplasmic reticulum Ca^2+^ leakage via RyR2 channels in cardiomyocytes leads to mitochondrial Ca^2+^ accumulation and dysfunction in heart failure (Santulli et al., 2015). In addition, RyR2 is an important molecular target of oxidative stress in cardiac myocytes, and RyR2 dysfunction could result in mitochondrial abnormalities and increased ROS production, dysmorphology and malfunction, which contributed to the pathogenesis of cardiac arrhythmia (Xie et al., 2015). In our study, we found that the myocardial mitochondrial structure was obviously destroyed, and the ATP content was decreased significantly. Thus, these findings suggested that disorders of calcium regulation in myocardial cells could lead to the abnormal structure of the mitochondria, resulting in myocardial cell function and energy metabolic disorders.

To further clarify the mechanism underlying the effects of *piggyBac* inserted into the RyR2 gene on mitochondrial structure and function, we compared the cardiac transcriptomes of RyR2-PBmice and WTmice and screened out a few of the differentially expressed genes closely related to RyR2 and mitochondrial function (see Dataset S7). In addition, by using RT-PCR and Western blot analysis, we found that the mRNA level of RyR2 in RyR2-PBmice cardiac tissue was decreased dramatically compared with that in WTmice, but the protein level of RyR2 had no significant difference between the two groups, which might be attributed to the post-transcriptional modification and could be an important reason why the RyR2-PBmice preserve a natural cardiac excitation-contraction coupling function.

In addition to RyR2, some other genes, such as GTP binding protein (Gs), Cyt C, Ucp3, mt-Atp, mt-Co2 and mt-Co3, were also downregulated at the transcriptional level, and these genes were involved in cardiomyocyte Ca^2+^ regulation and mitochondrial oxidation phosphorylation. Gs could phosphorylate the RyR2 by activating protein kinase A (PKA) (Izu et al., 2006; Sipido, 2007), and then, the protein structure of RyR2 was changed. Depending on the sensitivity of Ca^2+^ activation, the RyR2 channel opened, and Ca^2+^ was released from the endoplasmic reticulum to the cytosol (Carter, Colyer & Sitsapesan, 2006; Satoh et al., 2007). In RyR2-PBmice, the Gs decreased significantly at both the mRNA and protein levels, which could be an adaptive passivation response to the reduction of sarcoplasmic reticulum calcium capacity. The expression levels of ATPase, Cyt C and Ucp in cardiomyocytes all decreased, indicating that the electron transport chain might be destroyed, which was consistent with the observed mitochondrial structural abnormalities and dysfunction in RyR2-PBmice heart tissue.

Numerous studies have shown that the endoplasmic reticulum and mitochondria are the two organelles that are highly correlated in structure and function (Kohlhaas & Maack, 2010; Rutter & Pinton, 2014). Activation of the endoplasmic reticulum stress response has been shown to cause mitochondrial dysfunction (Mitchell et al., 2013), and mitochondrial oxidative stress further exacerbates the endoplasmic reticulum stress response (Tang et al., 2012). Therefore, we observed that in the RyR2-PBmouse heart tissue, the SR calcium capacity was significantly reduced, resulting in insufficient mitochondrial calcium intake and abnormal mitochondrial structure and function.

In conclusion, the animal model of RyR2-PBmice was successfully identified by tracking RFP and genotyping PCR. The homozygous RyR2-PBmice exhibited intrauterine death, while the heterozygotes survived well. Although the cardiac function in the heterozygous RyR2-PBmice did not show any abnormalities, the myocardial sarcoplasmic reticulum Ca^2+^ capacity decreased, and the expression levels of mitochondrial respiratory chain-related proteins were downregulated significantly, indicating that the insertion of *piggyBac* transposon into the RyR2 gene caused abnormal mitochondrial energy metabolism.

##  Supplemental Information

10.7717/peerj.6942/supp-1 Dataset S1 Heart rate of the WTmice and RyR2-PB mice (Fig. 3B) before and after the injection of caffeine and epinephrine

10.7717/peerj.6942/supp-2 Dataset S2 Statistic analysis of Fig. 4 Indexes of the phosphocreatine kinase (CK), lactate dehydrogenase (LDH), alanine aminotransferase (ALT) and aspartate aminotransferase (AST) in RyR2-PBmice and WTmice.

10.7717/peerj.6942/supp-3 Dataset S3 [Ca^2+^]_i_ transients in WTmice and RyR2-PBmice cardiomyocytes of Fig. 5 The basal and soproterenol (ISO) evoked [Ca^2+^]_i_ transients in WTmice and RyR2-PBmice cardiomyocytes, the fluorescence values (F) were normalized by the basal fluorescence (F_0_) in order to obtain the fluorescence ratio (F/F_0_).

10.7717/peerj.6942/supp-4 Dataset S4 Statistic analysis of Fig. 8 ATP concentration in myocardial tissue of WTmice and RyR2-PBmice.

10.7717/peerj.6942/supp-5 Dataset S5 Statistic analysis of Fig. 9 Quantitative RT-PCR analysis of RyR2 mRNA and the related signaling pathway coding sequences in WTmice and RyR2-PBmice.

10.7717/peerj.6942/supp-6 Dataset S6 Images of the western blots of Fig. 10 Western blot analysis of RyR2 and the related proteins regulating Ca^2+^ and ATP concentration in the heart tissues.

10.7717/peerj.6942/supp-7 Dataset S7 Cardiac transcriptome of the WTmice and RyR2-PBmice Raw data of the the differentially expressed genes in cardiac transcriptome between the WTmice and the RyR2-PBmice.

## Additional Information and Declarations

### Data Availability

The following information was supplied regarding data availability: The raw data is available as a Supplemental File.
